# Supplementary figures and images for: Induction of a chromatin boundary in vivo upon insertion of a TAD border
Source: PLoS Genet. 2021 Jul 22;17(7):e1009691. doi: 10.1371/journal.pgen.1009691 (PMC8330945; doi:10.1371/journal.pgen.1009691)

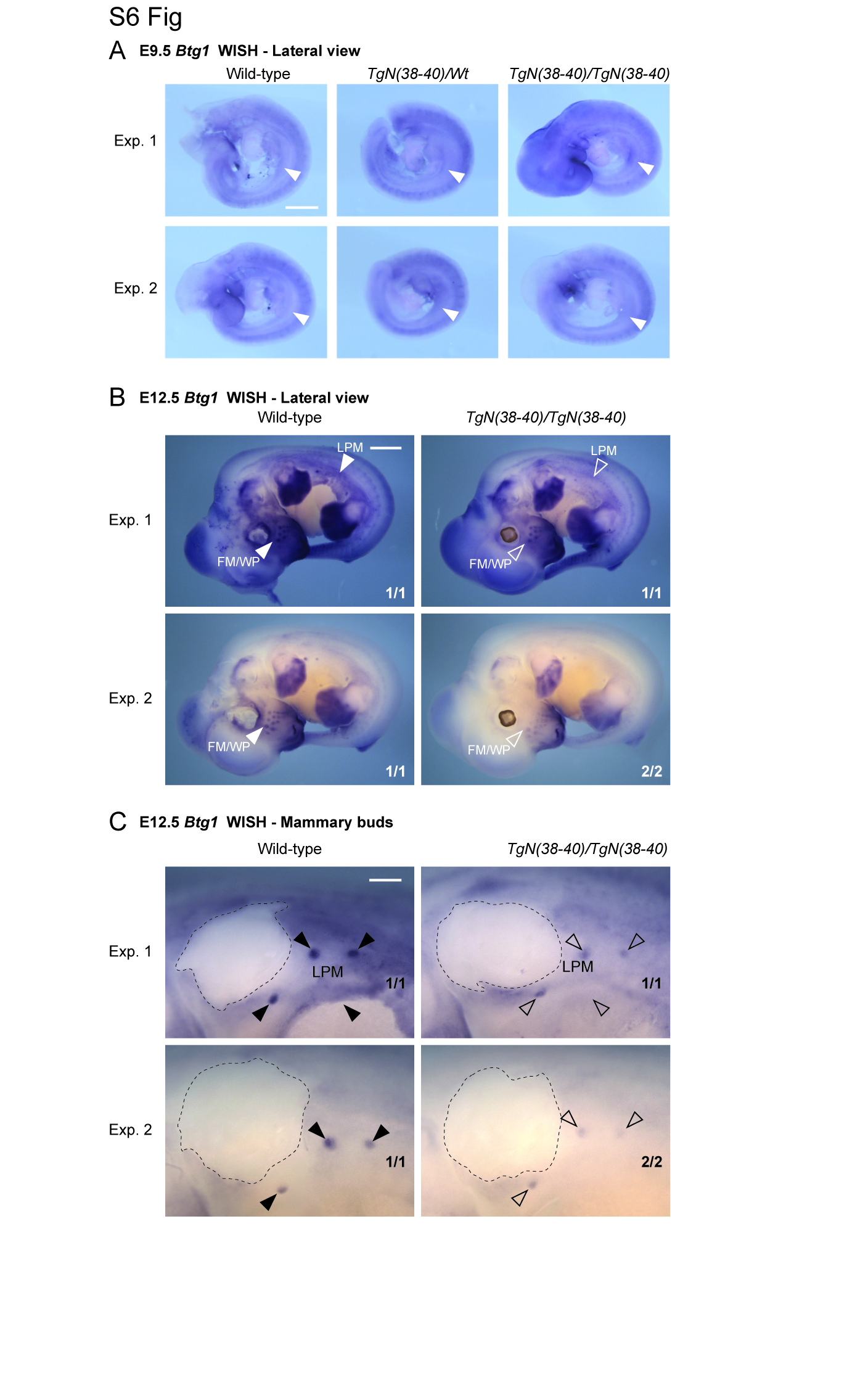

Supplement: S6 Fig — (A) Btg1 WISH in wild-type and TgN(38–40) mutant embryos at E9.5. Heads were partially severed and used for genotyping. Arrowheads point to the area where the presumptive limb bud is located. Scale bar: 500 μm. (B) Btg1 WISH in wild-type and TgN(38–40) homozygous embryos at E12.5. LPM: Lateral plate mesoderm. FM/WP: Facial mesenchyme and whisker pads. Scale bar: 1 mm. (C) Magnified pictures of mammary buds for the same embryos as in panel B. The position of the forelimbs, which were removed for easier mammary bud visualization, is highlighted by a dotted line. Scale bar: 300 μm. The proportion of embryos displaying equivalent patterns in each experiment is shown. Empty arrowheads indicate changes in expression compared to bold arrowheads. (TIF) [file pgen.1009691.s006.tif]
