# Supplementary material for: Induction of a chromatin boundary in vivo upon insertion of a TAD border
Source: PLoS Genet. 2021 Jul 22;17(7):e1009691. doi: 10.1371/journal.pgen.1009691 (PMC8330945; doi:10.1371/journal.pgen.1009691)
Supplement: S1 Table — Source data (for different genotypes and mapped genomes) and TAD calling window sizes (w) are indicated. At the HoxD locus, Atf2 is the left boundary of the C-DOM and Hnrnpa3 is the right boundary of the T-DOM. Left_Bd and Right_Bd are respectively the left and right boundaries of the TAD hosting the TgN(38–40) construct in chromosome 10 (see Figs 3B and 4A). (DOCX) [file pgen.1009691.s007.docx]

**S1 Table**

| **Boundary**  **Data**  **TAD calling w** | ***Atf2*** | ***HoxD*** | **CS38-40** | ***Hnrnpa3*** | **Left_Bd** | **Integration** | **Right_Bd** |
| --- | --- | --- | --- | --- | --- | --- | --- |
| **Wt_map_mm10**  **240kb** | YES | YES | YES | YES | YES | NO | YES |
| **Wt_map_mm10**  **320kb** | YES | YES | NO | YES | YES | NO | YES |
| **Wt_map_mm10**  **480kb** | NO | YES | NO | YES | YES | NO | YES |
| **Wt_map_mm10**  **800kb** | NO | YES | NO | NO | YES | NO | YES |
| **Wt_map_Tg+del**  **240kb** | YES | YES | NO | YES | YES | NO | YES |
| **Wt_map_Tg+del**  **320kb** | YES | YES | NO | YES | YES | NO | YES |
| **Wt_map_Tg+del**  **480kb** | NO | YES | NO | YES | YES | NO | YES |
| **Wt_map_Tg+del**  **800kb** | NO | YES | NO | NO | YES | NO | YES |
| **Tg+del_map_mm10**  **240kb** | YES | YES | NO | YES | YES | YES | YES |
| **Tg+del_map_mm10**  **320kb** | YES | YES | NO | YES | YES | YES | NO |
| **Tg+del_map_mm10**  **480kb** | NO | YES | NO | YES | YES | YES | NO |
| **Tg+del_map_mm10**  **800kb** | NO | YES | NO | NO | YES | NO | YES |
| **Tg+del_map_Tg+del**  **240kb** | YES | YES | NO | YES | YES | YES | YES |
| **Tg+del_map_Tg+del**  **320kb** | YES | YES | NO | YES | YES | YES | NO |
| **Tg+del_map_Tg+del**  **480kb** | NO | YES | NO | YES | YES | NO | YES |
| **Tg+del_map_Tg+del**  **800kb** | NO | YES | NO | NO | YES | NO | YES |

**S1 Table.** Identification of topological boundaries using various window sizes. Source data (for different genotypes and mapped genomes) and TAD calling window sizes (w) are indicated. At the *HoxD* locus, *Atf2* is the left boundary of the C-DOM and *Hnrnpa3* is the right boundary of the T-DOM. Left_Bd and Right_Bd are respectively the left and right boundaries of the TAD hosting the TgN(38-40) construct in chromosome 10 (see Figs 3B and 4A).
