# Supplementary material for: Induction of a chromatin boundary in vivo upon insertion of a TAD border
Source: PLoS Genet. 2021 Jul 22;17(7):e1009691. doi: 10.1371/journal.pgen.1009691 (PMC8330945; doi:10.1371/journal.pgen.1009691)
Supplement: S2 Table — For the 4C-seq primers, Illumina Solexa sequencing adapters are indicated in red (long adapter) or blue (short adapter). For both CS38 and CS40 viewpoints, a 4 bp barcode (underlined) was present between the long sequencing adapter and the rest of the primer. F: forward. R: reverse. iF: inverse forward. iR: inverse reverse. (DOCX) [file pgen.1009691.s008.docx]

**S2 Table**

| **Primer** | **Sequence** | **Use** |
| --- | --- | --- |
| TLA-CS38_iF | GGGCAGAACTCACTGATTAA | TLA |
| TLA-CS38_iR | ATCATTTGGGGATTGCAGAA | TLA |
| TLA-vector_iF | TCACAGGTATTTATTCGCGA | TLA |
| TLA-vector_iR | TTGTTACACCGTTTTCCATG | TLA |
| qPCR-CS38_F | GCAAAGCTCGACCTACACTTC | qPCR |
| qPCR-CS38_R | ATTTACAGTGCGAGGGTCTCC | qPCR |
| qPCR-CS39_F | TCACGCTGACACTGTGTTTG | qPCR |
| qPCR-CS39_R | GCAGGTTACGTAATGTGTGCTG | qPCR |
| qPCR-CS40a_F | TGGTGGTGAAGAAGCGGTAAG | qPCR |
| qPCR-CS40a_R | CGACAGCCTTTCCATCTGTTTG | qPCR |
| qPCR-Hoxd8d9_F | TCAGCGGTCCAAACCCAAGTCA | qPCR |
| qPCR-Hoxd8d9_R | CTGCACCACAACGCTAGCTTTAC | qPCR |
| qPCR-Aldh1a2_F | CTCTTTGGCCATAAACGTTCCC | qPCR |
| qPCR-Aldh1a2_R | TAAAGTGACCGAGCAAGCAC | qPCR |
| 4C-CS38_iF | AATGATACGGCGACCACCGAACACTCTTTCCCTACACGACGCTCTTCCGATCTATCGTTCCAAGGAGAAAGGTGTTGGTC | 4C-seq |
| 4C-CS38_iR | CAAGCAGAAGACGGCATACGACAGGGCGTTGGGTCACTCT | 4C-seq |
| 4C-CS40_iF | AATGATACGGCGACCACCGAACACTCTTTCCCTACACGACGCTCTTCCGATCTGCCAAACATTTTCCTGCTTCTTAGTC | 4C-seq |
| 4C-CS40_iR | CAAGCAGAAGACGGCATACGAAAGCAAGACACAGAGAGATG | 4C-seq |
| 4C-CTCF-left_iF | AATGATACGGCGACCACCGAACACTCTTTCCCTACACGACGCTCTTCCGATCTGTTCCAGAATGTCCCAGG | 4C-seq |
| 4C-CTCF-left_iR | CAAGCAGAAGACGGCATACGATATCAGCCCCAGAGTAGATC | 4C-seq |
| 4C-CTCF-right_iF | AATGATACGGCGACCACCGAACACTCTTTCCCTACACGACGCTCTTCCGATCTACGTTTATAGTCTAATTGGGCA | 4C-seq |
| 4C-CTCF-right_iR | CAAGCAGAAGACGGCATACGAATTGTGTTTTCGGTTGCTTT | 4C-seq |
| RT-qPCR_Btg1_F | gtgtccttcatctccaagttcc | RT-qPCR |
| RT-qPCR_Btg1_R | TAATGTTctgccagcagctc | RT-qPCR |
| RT-qPCR_Tbp_F | CTACCGTGAATCTTGGCTGTAAAC | RT-qPCR |
| RT-qPCR_Tbp_R | AATCAACGCAGTTGTCCGTGGC | RT-qPCR |
| RT-qPCR_Actb_F | CATTGCTGACAGGATGCAGAAGG | RT-qPCR |
| RT-qPCR_Actb_R | TGCTGGAAGGTGGACAGTGAGG | RT-qPCR |

**S2 Table.** List of TLA, qPCR, 4C-seq and RT-qPCR primers used in this study. For the 4C-seq primers, Illumina Solexa sequencing adapters are indicated in red (long adapter) or blue (short adapter). For both CS38 and CS40 viewpoints, a 4 bp barcode (underlined) was present between the long sequencing adapter and the rest of the primer. F: forward. R: reverse. iF: inverse forward. iR: inverse reverse.
