# Supplementary material for: Induction of a chromatin boundary in vivo upon insertion of a TAD border
Source: PLoS Genet. 2021 Jul 22;17(7):e1009691. doi: 10.1371/journal.pgen.1009691 (PMC8330945; doi:10.1371/journal.pgen.1009691)
Supplement: S3 Table — The name of the oligos indicates their approximate position on the TgN(38–40) transgene or surrounding regions: upstream of the transgene in chr 10 (up); one quarter (1/4), halfway (1/2) or three quarters (3/4) into the transgene; 3’ part of transgene vector (pEpi3) and downstream of the transgene in chr10 (down). Two pairs of primers (1 or 2) were design to target the same region. Underlined, sequence matching target DNA. Red, sequence of the T7 promoter for sgRNA production. Cyan, RNA scaffold for the Cas9 enzyme. A G was added (highlighted in black) when not present in the original target sequence, to ensure efficient sgRNA transcription. (DOCX) [file pgen.1009691.s009.docx]

**S3 Table**


| **EnGen-compatible oligo** | **Sequence** |
| --- | --- |
| EnGen_up_1 | TTCTAATACGACTCACTATAGAGATGGTGAAAATGACCTGGGTTTTAGAGCTAGA |
| EnGen_up_2 | TTCTAATACGACTCACTATAGAGATTTGTTTGGGAGTGCGGGTTTTAGAGCTAGA |
| EnGen_1/4_1 | TTCTAATACGACTCACTATAGTGGTGCAGGAGATACACAGAGTTTTAGAGCTAGA |
| EnGen_1/4_2 | TTCTAATACGACTCACTATAGACTAGTAAAACAACTTGATGGTTTTAGAGCTAGA |
| EnGen_1/2_1 | TTCTAATACGACTCACTATAGTGGTAATCTAGCAAGCTCTGGTTTTAGAGCTAGA |
| EnGen_1/2_2 | TTCTAATACGACTCACTATAGAGTGCATAGAATCCCCAAAGGTTTTAGAGCTAGA |
| EnGen_3/4_1 | TTCTAATACGACTCACTATAGTGATGGCTAGGTCACAGGAGTTTTAGAGCTAGA |
| EnGen_3/4_2 | TTCTAATACGACTCACTATAGCTAGAAAGAGAAACCACAGAGTTTTAGAGCTAGA |
| EnGen_pEpi3_1 | TTCTAATACGACTCACTATAGCTAGTGATAATAAGTGACTGGTTTTAGAGCTAGA |
| EnGen_pEpi3_2 | TTCTAATACGACTCACTATAGCAAGAAGAAATATCCACCGGTTTTAGAGCTAGA |
| EnGen_down_1 | TTCTAATACGACTCACTATAGATGTGTATTGCAGAAGTCAGGTTTTAGAGCTAGA |
| EnGen_down_2 | TTCTAATACGACTCACTATAGTAAATAAAATTTATAATCTGGTTTTAGAGCTAGA |


**S3 Table.** EnGen-compatible DNA oligos used as templates for sgRNA production. The name of the oligos indicates their approximate position on the *TgN(38-40)* transgene or surrounding regions: upstream of the transgene in chr 10 (up); one quarter (1/4), halfway (1/2) or three quarters (3/4) into the transgene; 3' part of transgene vector (pEpi3) and downstream of the transgene in chr10 (down). Two pairs of primers (1 or 2) were design to target the same region. Underlined, sequence matching target DNA. Red, sequence of the T7 promoter for sgRNA production. Cyan, RNA scaffold for the Cas9 enzyme. A G was added (highlighted in black) when not present in the original target sequence, to ensure efficient sgRNA transcription.
