# Supplementary material for: Induction of a chromatin boundary in vivo upon insertion of a TAD border
Source: PLoS Genet. 2021 Jul 22;17(7):e1009691. doi: 10.1371/journal.pgen.1009691 (PMC8330945; doi:10.1371/journal.pgen.1009691)
Supplement: S4 Table — Target reads are reads mapping to the construct or the integration site. Construct coordinates taken into consideration: chr2:75123000–75160000 (mm10). Considered integration site: chr10:97018700–97019300 (mm10). *Ratio of bases of the mutant construction (64,420 bp; see Fig 2D) relative to the haploid mouse genome (around 2.6 Gb): 2.457384e-05. (DOCX) [file pgen.1009691.s010.docx]

**S4 Table**


| **MinION output summary** | |
| --- | --- |
| Longest mapped read | 76,241 bp |
| N50 | 7,610 bp |
| Longest target read | 36,312 bp |
| N50 of target reads | 9,878 bp |
| ***Reads above 0 kb (all reads)*** | |
| Total reads | 776,913 |
| Target reads | 433 |
| Reads fully mapped on the mutant construction | 248 out of 776,913  (ratio 3.192121e-04) |
| Bases fully mapped on the mutant construction | 1,654,070 out of 2,823,381,905 (ratio 5.858471e-04) |
| Enrichment of bases fully mapped on the mutant construction  (5.858471e-04/2.457384e-05*) | 23.84028 |
| ***Reads above 2 kb*** | |
| Total reads | 372,655 |
| Target reads | 240 |
| Reads fully mapped on the mutant construction | 180 out of 372,655  (ratio 4.830205e-04) |
| Bases fully mapped on the mutant construction | 1,583,862 out of 2,495,725,562 (ratio 6.346299e-04) |
| Enrichment of bases fully mapped on the mutant construction  (6.346299e-04/2.457384e-05*) | 25.82543 |


**S4 Table.** Summary of the MinION sequencing output. Target reads are reads mapping to the construct or the integration site. Construct coordinates taken into consideration: chr2:75123000-75160000 (mm10). Considered integration site: chr10:97018700-97019300 (mm10). *Ratio of bases of the mutant construction (64,420 bp; see Fig 2D) relative to the haploid mouse genome (around 2.6 Gb): 2.457384e-05.
