# Supplementary material for: Induction of a chromatin boundary in vivo upon insertion of a TAD border
Source: PLoS Genet. 2021 Jul 22;17(7):e1009691. doi: 10.1371/journal.pgen.1009691 (PMC8330945; doi:10.1371/journal.pgen.1009691)
Supplement: S5 Table — Two samples were sequenced: TgN3840 (mutant) and Wt (control). Both were subsequently mapped either on mm10 wild-type mouse genome or the custom TgN(38–40) genome. Total reads correspond to all raw reads obtained from the sequencing platform. Cis-far reads correspond to intra-chromosomal interactions located further than 10 kb. All sequencing outputs are shown as base pairs (bp). (DOCX) [file pgen.1009691.s011.docx]

**S5 Table**

| **Sample** | **Total reads** | **Paired read** | **Valid pairs** | **Deduplication unique read pairs** | **Deduplication unique cis-far reads** |
| --- | --- | --- | --- | --- | --- |
| E12 Limbs TgN3840 map mm10 | 96,383,826 | 54,092,058 | 52,976,891 | 51,582,332 | 32,235,636 |
| E12 Limbs TgN3840 map TgN3840 | 96,383,826 | 81,045,553 | 79,748,405 | 78,059,314 | 40,600,249 |
| E12 Limbs Wt  map mm10 | 140,552,702 | 80,733,773 | 78,828,076 | 76,191,470 | 49,239,923 |
| E12 Limbs Wt  map TgN3840 | 140,552,702 | 119,258,173 | 117,078,012 | 113,895,884 | 61,080,153 |


**S5 Table.** Summary of the Hi-C sequencing output. Two samples were sequenced: TgN3840 (mutant) and Wt (control). Both were subsequently mapped either on mm10 wild-type mouse genome or the custom TgN(38-40) genome. Total reads correspond to all raw reads obtained from the sequencing platform. Cis-far reads correspond to intra-chromosomal interactions located further than 10 kb. All sequencing outputs are sown as base pairs (bp).
