# Supplementary material for: Induction of a chromatin boundary in vivo upon insertion of a TAD border
Source: PLoS Genet. 2021 Jul 22;17(7):e1009691. doi: 10.1371/journal.pgen.1009691 (PMC8330945; doi:10.1371/journal.pgen.1009691)
Supplement: S6 Table — (DOCX) [file pgen.1009691.s012.docx]

**S6 Table**

| **Viewpoint name and genotype (in italics)** | **Total reads** | **Mapped reads** | **Proportion of mapped reads** |
| --- | --- | --- | --- |
| E12 Limbs *Wt* CTCF-right | 3,280,736 | 2,868,671 | 87.44 |
| E12 Limbs *TgN3840* CTCF-right | 2,996,923 | 2,603,075 | 86.86 |
| E12 Limbs *TgN3840* CS40 | 3,650,116 | 3,363,099 | 92.14 |
| E12 Limbs *Wt* CTCF-left | 3,857,330 | 3,216,376 | 83.38 |
| E12 Limbs *TgN3840* CTCF-left | 4,323,410 | 3,570,129 | 82.58 |
| E12 Limbs *TgN3840*_CS38 | 3,305,420 | 2,947,165 | 89.16 |

**S6 Table**. Summary of mapped reads from 4C-seq experiments.
