# Supplementary material for: Induction of a chromatin boundary in vivo upon insertion of a TAD border
Source: PLoS Genet. 2021 Jul 22;17(7):e1009691. doi: 10.1371/journal.pgen.1009691 (PMC8330945; doi:10.1371/journal.pgen.1009691)
Supplement: S7 Table — (DOCX) [file pgen.1009691.s013.docx]

**S7 Table**

| File name | Number of fragments | % of trans-interactions | Signal in plotted regions | Number of plotted fragments |
| --- | --- | --- | --- | --- |
| segToFrag_E12_Limbs_TgN3840_CS40.bw | 27,133 | 80.95 | 9.31 | 1196 |
| segToFrag_E12_Limbs_TgN3840_CS38.bw | 36,895 | 73.27 | 14.55 | 1648 |
| segToFrag_E12_Limbs_TgN3840_CTCF-right.bw | 20,821 | 72.78 | 14.77 | 1274 |
| segToFrag_E12_Limbs_Wt_CTCF-right.bw | 21,242 | 71.71 | 17.87 | 1419 |
| segToFrag_E12_Limbs_TgN3840_CTCF-left.bw | 33,374 | 75.03 | 15.25 | 1476 |
| segToFrag_E12_Limbs_Wt_CTCF-left.bw | 33,257 | 74.52 | 16.16 | 1539 |

**S7 Table**. Summary of 4C-seq fragment distribution.
