# Supplementary material for: Induction of a chromatin boundary in vivo upon insertion of a TAD border
Source: PLoS Genet. 2021 Jul 22;17(7):e1009691. doi: 10.1371/journal.pgen.1009691 (PMC8330945; doi:10.1371/journal.pgen.1009691)
Supplement: S8 Table — Wild-type ChIP-seq data of CTCF, RAD21, and H3K27ac were retrieved from a previous publication of our group (see Data availability). WL: whole limbs. DFL: distal forelimbs. (DOCX) [file pgen.1009691.s014.docx]

**S8 Table**

| **Experiment** | **Genotype** | **Tissue** | **Biological replicates** |
| --- | --- | --- | --- |
| CTCF ChIP-seq | *Wild-type* | E12.5 WL | 1 |
| CTCF ChIPm | *TgN(38-40)/Wt;*  *del(CS38-40)^-/-^* | E12.5 WL | 2 |
| RAD21 ChIP-seq | *Wild-type* | E12.5 WL | 1 |
| RAD21 ChIPm | *TgN(38-40)/Wt;*  *del(CS38-40)^-/-^* | E12.5 WL | 2 |
| H3K27ac ChIP-seq | *Wild-type* | E12.5 DFL | 1 |

**S8 Table.** Biological replicates of the ChIP-seq and ChIPmentation (ChIPm) experiments. *Wild-type* ChIP-seq data of CTCF, RAD21, and H3K27ac were retrieved from a previous publication of our group (see Data availability). WL: whole limbs. DFL: distal forelimbs.
