# Supplementary material for: Induction of a chromatin boundary in vivo upon insertion of a TAD border
Source: PLoS Genet. 2021 Jul 22;17(7):e1009691. doi: 10.1371/journal.pgen.1009691 (PMC8330945; doi:10.1371/journal.pgen.1009691)
Supplement: S9 Table — Genotypes of 4C-seq samples are colored in the same way than the corresponding tracks of Fig 3. WL: whole limbs (including both forelimbs and hindlimbs). (DOCX) [file pgen.1009691.s015.docx]

**S9 Table**

| **Experiment** | **Viewpoint** | **Genotype** | **Tissue** |
| --- | --- | --- | --- |
| 4C-seq | CS38 | *TgN(38-40)/TgN(38-40)* | E12.5 WL |
|  | CS40 | *TgN(38-40)/TgN(38-40)* | E12.5 WL |
|  | CTCF-left | *Wt/Wt* | E12.5 WL |
|  |  | *TgN(38-40)/TgN(38-40)* | E12.5 WL |
|  | 3’ *Btg1* | *Wt/Wt* | E12.5 WL |
|  |  | *TgN(38-40)/TgN(38-40)* | E12.5 WL |
|  | CTCF-right | *Wt/Wt* | E12.5 WL |
|  |  | *TgN(38-40)/TgN(38-40)* | E12.5 WL |
| Hi-C | Not applicable | *Wt/Wt* | E12.5 WL |
|  |  | *TgN(38-40)/TgN(38-40); del(CS38-40)^-/-^* | E12.5 WL |

**S9 Table.** Genotypes of 4C-seq and Hi-C samples. Genotypes of 4C-seq samples are colored in the same way than the corresponding tracks of Fig 3. WL: whole limbs (including both forelimbs and hindlimbs).
