# Supplementary material for: Induction of a chromatin boundary in vivo upon insertion of a TAD border
Source: PLoS Genet. 2021 Jul 22;17(7):e1009691. doi: 10.1371/journal.pgen.1009691 (PMC8330945; doi:10.1371/journal.pgen.1009691)
Supplement: Attachment — Submitted filename: Willemin_2021_PLoS_Genetics_response_to_referees_20210609.docx [file pgen.1009691.s017.docx]

**Manuscript ID: PGENETICS-D-21-00201**

Title from initial submission: "CONTEXT-INDEPENDENT FUNCTION OF A CHROMATIN BOUNDARY *IN VIVO*"

Andréa Willemin^a,1,†^, Lucille Lopez-Delisle^b,1^, Christopher Chase Bolt^b^, Marie-Laure Gadolini^a^, Denis Duboule^a,b,c,2,#^, and Eddie Rodríguez-Carballo^a,2,‡,#^

RESPONSES TO REFEREES

Dear Editors,

We are grateful to the reviewers for their insightful comments and suggestions. We have taken into consideration all their inputs and we address them the best we can below. For the ease of reading, we have copied their comments *in extenso* in italics and our answers are to be found below each item. In addition, and whenever this was possible, we have adapted figures in response to the recommendations.

Thank you for handling our manuscript and offering us to submit a revised version.

Best wishes,

Denis Duboule

Eddie Rodríguez-Carballo

**List of changes in the Figures and the Manuscript**:

In the Manuscript:

*The title has been changed.

*The present address of Andréa Willemin was corrected.

*The main text and figure legends were adapted to accommodate the new results and figures, as well as in response to the comments of the reviewers.

*The discussion was amended to include the RT-qPCR results. Also, the Huang et al. 2020 paper was incorporated to the discussion.

In the Figures:

Figure 1:

*Panel A, B: TAD horizontal bars are now coloured.

*Panel C, new: The TAD isolation profiles are now added to the figure.

*Panel D, E: Re-labelling of former panels C and D .

Figure 3:

*Panel A: horizontal dotted lines were added to help comparing between the two CTCF tracks.

*Panel B: "Left_Bd and "Right_Bd" are now "Left Bd" and "Right Bd". The horizontal bars representing the TADs are now coloured.

Figure 4:

*Panels B and C: TAD horizontal bars are coloured. Hi-C of wt is now adapted to correct for distance bias according to the value of alpha of the wt *Btg1* TAD.

*Panel D: The legend has been modified to include "gain" and "loss". ENCODE ChIP-seq tracks of E12.5 liver and limbs are now incorporated into the figure.

*Panel E: The second replicate of *Btg1* WISH has been removed.

*Panel F: A new panel has been added showing qPCR of *Btg1* in distal limb and liver cells of mutant and wild-type embryos.

*Panel G: Re-labelling (former panel F).

Figure S4 (novel):

* Panels A, B, C: CTCF ChIP tracks of regions unrelated to *Btg1* or *HoxD* loci.

Figure S5 (novel):

* Panel A: Frequencies of interactions as a function of genomic distance.

* Panel B: Hi-C over the first TADs of chromosome 10 used in panel A.

Figure S6 (formerly Fig S4).

*Panel A (novel): E9.5 RNA WISH of *Btg1*.

*Panel B, C: Formerly panels A and B, respectively.

Tables

Table S2 now incorporates the RT-qPCR primers.

Table S5 (novel) contains sequencing statistics from Hi-C experiments.

Tables S6 and S7 (novel) show different 4C-seq information regarding mapped reads and fragment distribution.

Tables S8 and S9 are former Table S5 and S6, respectively.

Table S10. RT-qPCR values from E12.5 distal forelimbs and livers from wild-type and heterozygous (*TgN(CS38-40)*/Wt) embryos. *Btg1* mRNA is the target gene. *Actb* is used as reference control and *Tbp* as an unchanged internal control.

***Reviewer #1:***

*This is an interesting study reporting the first accurate characterization of the in vivo structural and functional effects of an ectopic insertion of genomic sequence containing a TAD boundary. The experiments meet high technical standards and in addition to providing interesting insight into the role of chromosome topology, also highlight the fact that every ectopic insertion deserves very careful examination before its structural and functional impact can be assessed.*

*The main limitation of the manuscript is that only one ectopic location and one version of the ectopic sequence are analyzed. This makes it hard to infer how generally the results can be extrapolated, and also to formally conclude on the mechanistic role of CTCF sites and loop extrusion in establishing the interactions observed at the ectopic location. I do not think however that this should be held up against the manuscript, given the complexity and timescales of in vivo experiments.*

We certainly agree on this point and indeed we present data coming from one single integration event, which is a limitation of our study. For this reason, however, we believe we are careful not to draw too broad and general conclusions that would apply to all boundary insertions. In fact, we did analyse series of other insertions, yet in many cases the insertion of the transgene had not occurred as anticipated (mostly in copy number, etc). We think that this is not specific to this study but that previous studies of this kind have only superficially evaluated the modalities of transgene insertions. This is the reason why we report only one fully determined insertion site and also why we have introduced a sentence in the discussion warning about the use of such transgenic approaches and the necessity for a complete analysis of the transgene on site

Regarding the role of CTCF in this particular insertion, we also think that the best control would be to modify the sequence of the inserted transgene. As reviewer #1 points out, performing this approach *in vivo* would be outside any reasonable time frame.

*I only have a very small number of comments and suggestions (#1 is crucial though):*

*1) I would like to point the authors to the fact that the differential analysis of Hi-C data (Fig. 4) is not formally correct. Simply inserting a blank space in the WT map does not account for the correct decay of contact probabilities that would be generated by ~60kb of extra genomic sequence, even in the complete absence of CTCF sites or any other sequences that can create specific interactions. This is due to the power-law behavior of contact probabilities and 60 kb can make a big difference!*

*In order to conclude that sequence identity (rather than length) is responsible for the appearance of an extra sub-TAD boundary, the transgenic sample should be compared to a better (although not perfect) control where the WT counts across the insertion are rescaled by the average power-law decay inside the original TAD, which can be extracted from the WT Hi-C map. The formally correct control, which however seems out of experimental reach in the context of this study, would be a line carrying the homozygous insertion of the same ectopic sequence without CTCF sites.*

We have thoroughly considered the comment of reviewer #1. We agree with her/his opinion regarding the presumptive consequence of the extra genomic length into this locus of chromosome 10. As mentioned, the idea of generating homozygous mutants without the CTCF binding sites was not pursued. Nevertheless, and to comply with this issue, we performed a genome-wide analysis of all interactions in our wild-type limbs Hi-C dataset and we confirmed that they follow a power-law decay with increasing genomic distances (see below Figure R1A, red lines, alpha = -1.01), as found in multiple reports (reviewed in Pombo and Nicodemi, 2014 and Lajoie et al 2015, for examples).

In order to determine whether this power-law decay also took place within TADs, we performed the same analysis but restricted the results to the extension of all TADs and, as show in Fig R1A (black lines), there is still a strong power-law decay with a slightly higher value of alpha (-0.84). We then focused our analysis on the *Btg1* TAD and noticed that again the frequency of contacts decreases when distance increases (Fig R1A, pink lines). However, this behavior did not seem to be a simple power-law (R² = 0.82) and the fitted value of alpha (-0.42) is much higher than the one obtained with the two other conditions. We then aimed to see if the behaviour of the *Btg1* TAD was referable to other discrete TADs, instead of comparing it to broader analysis (genome-wide and all-TADs aggregated). We analysed the first eight TADs of chromosome 10 (Figure R1B below, mm10 chr10:3960000-9040000) and plotted the contacts-to-distance relationship of the six most reliable TADs. We observed that, although contact frequency tended to decrease along distance, individually, TADs did not behave in a uniform manner (see TAD1-8 in Fig. R1A) and were mostly affected in the contacts (loop) established at each TAD border.

Legend to Figure R1*.* (A) Analysis of interaction frequencies against genomic distances. Bold lines show the real distribution of the relationship whereas the dashed line is the fitted correlation. The value of alpha and the R-squared values for each analysed genomic stretch are shown. The plot includes a genome-wide analysis, an aggregate curve of all TADs, the *Btg1* TAD and six of the first TADs of chromosome 10 (mm10 chr10:3960000-9040000). (B) Hi-C matrix of the first eight TADs of chromosome 10. (C) Analysis of contact distribution of each 20 kb bin (grey lines) located in the genomic area marked as a dashed box. The bold black line represents the aggregate of all the combined bins. CTCF binding sites and their orientations are shown as blue and red triangles. The extension of each TAD is shown as horizontal bars below each graph (light blue for wt and light red for TgN(38-40).

In addition and given that the *Btg1* TAD is the focus of our study, we wanted to visualize bin per bin if we could observe a clear influence of distance in the distribution of interaction frequencies, especially in the dashed region of Fig. 4C. As shown in Fig. R1C, we plotted the contact frequencies of each 20 kb bin located in a 5' (left) region of the *Btg1* TAD. In order to avoid noise in the signal due to the proximity between the bins and the insertion site, we restricted the analysis to bins located more than 120 kb apart from the integration (dashed box in Fig. R1C). To assess if the use of any mapping strategy (meaning the genome used to map the data) could bias the interpretation, we carried out the above-mentioned approach with the wt dataset mapped on mm10, the wt dataset mapped on the custom TgN(38-40) genome (as in Fig4) and the mutant TgN(38-40) data mapped on its respective genome. None of the wt plots showed any clear decay of contact frequencies with the 3' part of the *Btg1* TAD, and the contacts with the right TAD border were evident and comparable in both cases. Notwithstanding, in the case of the mutant dataset, the average contact frequencies after the integration site were clearly diminished, thus confirming that the insertion of the transgene did affect the interactions across this locus.

This analysis confirmed that the extra genomic distance did not dramatically bias our results and that our initial interpretation was correct. We are thankful to the reviewer for raising this point since it allowed to re-enforce the validity of our conclusions. Consequently, we have applied the correction proposed by the reviewer, using the value of alpha of the *Btg1* wt TAD (alpha = -0.42) as found in Fig. R1A. A new version of Figure 4 has been produced with the correction applied to the wt sample. To illustrate and justify the normalization of the mutant Hi-C dataset in the manuscript, we have integrated part of Fig. R1 as a new supplementary Figure (S5).

Furthermore, we attach below a table (Table R1) including the statistical analysis before and after the proposed correction, showing similar values.

| Table R1 | Before correction | After correction (α= -0.42) |
| --- | --- | --- |
| Average contact frequency | 0.0130 | 0.0124 |
| Loss of interaction | -42% | -39% |
| p-value | 2e-32 | 2e-29 |

*2) In Fig. 4C it is unclear what the scalebar represents.*

We have adapted the figure and simplified the legend by stating "gain" and "loss" of interactions.

*3) It would be very interesting to explore to which extent the amount of Btg1 downregulation depends on the amount of physical insulation provided by the ectopic boundary. For example does it depend on the number of CTCF sites in the boundary, as recently reported (Huang et al.,* [*https://www.biorxiv.org/content/10.1101/2020.07.07.192526v1*](https://www.biorxiv.org/content/10.1101/2020.07.07.192526v1)*)? The homozygous TgN(38-40) line provides an ideal background for generating CRISPR deletions affecting the number of CTCF sites in the transgenic boundary.*

As mentioned above, we agree that the proposed experiment would better characterize the contribution of the number of CTCF binding sites to the insulation potential of this boundary and its impact upon *Btg1* activation. Nevertheless, generating the appropriate combinations of CTCF deletions *in vivo* would take at least another year of work, *a fortiori* in a direction that we do not want to pursue.

*4) It would be nice to see higher magnification Hi-C maps – it looks like the number of reads in the experiments is not reported (should be included in a revised version) but from a qualitative assessment of the maps provided, this could be feasible.*

Following this suggestion, the number of reads obtained from the Hi-C experiments are now reported as Supplementary Table 5.

Also, we have mapped the Hi-C datasets binned at 20 kb but the resolution was not high enough to obtain visually satisfactory results; besides, TAD calling appeared less predictable (see arrow in Fig. R2B).

In Rao et al. (2014), the authors defined the "map resolution" as the smallest locus size such that 80% of loci have at least 1,000 contacts. We thus computed the proportion of bins displaying at least 1,000 intra-chromosomal contacts. We found around 90% for the 40 kb bins followed this trend, but only around 30% of the 20 kb bins, confirming that 40 kb was the bin size best representing our data.

Legend to Figure R2*.* A different version of main Fig. 1 (A, B) Hi-C maps of wt and *del(CS38-40)* binned at 20 kb resolution. In panel B, an arrow points to an artefactual new TAD boundary inside T-DOM detected in the *del(CS38-40)* dataset.

**Reviewer #2:**

*In this work the authors use mouse transgenesis to randomly insert a previously characterized TAD subdomain boundary from the HoxD locus randomly into the mouse genome. First, the authors in detail characterize the transgene insertion in the Btg1 locus using a elaborate combination of state of the art approaches and find that the boundary to be inserted in a partial tandem copy within the Btg1 TAD. Following this, the authors characterize the effects of the inserted boundary on 3D chromatin structure and find that also at the new location the boundary element consisting of 4 CTCF sites (3+1 duplicated) serves as a sub-domain boundary (and not a "full/strong" boundary) that allows some contacts beyond the insertion point. Using in situ hybridizations of E12.5 embryos the authors show that the insertions leads to an overall reduced expression level of Btg1 without major changes in expression pattern. The in-depth analysis of the transgene insertion using multiple analogous methods can serve as a case example of how to comprehensively characterize new transgenes, especially for studies that "assume" that the knock-in happened as predicted in theory.*

*The characterization of the 3D chromatin effects that originate from the sub-domain boundary are interesting, although limited in scope, given that only one alternative boundary position is analyzed. Stik et al in HAP1 cells and Huang et al (*[*https://www.biorxiv.org/content/10.1101/2020.07.07.192526v1*](https://www.biorxiv.org/content/10.1101/2020.07.07.192526v1)*) in mouse embryonic stem cells have taken an alternative approach and characterized several integrations in cell culture systems. However, the in vivo approach taken here enables a more comprehensive functional readout at an organismal level similar to our own boundary repositioning at the Sox9 locus.*

*Therefore, the characterization of the gene regulatory effects of the boundary insertion should be more comprehensive in order to fully realize the potential of in vivo approach that the authors undertook. I would suggest including additional data that should be available or easily attainable to the authors (if the mouse line is still available) prior to publication.*

*Major comments:*

*Regulatory effects 1:*

*The authors should expand the regulatory consequences of the CS38-40 insertion into the Btg1 domain on Btg1 expression. As the authors have previously shown (Andrey et al 2013, Rodriguez-Carballo et al 2020), CS39 comprises a potent limb bud enhancer that is particularly active at stages prior to E12.5 and the TSS of two lncRNAs. If the mouse lines are available, it would be very informative to determine whether this leads to increased Btg1 levels at these early stages, assessed through WISH, but preferably also through more quantitative methods. Also, do lncRNA transcripts arise from the knock-in construct?*

To assess if the transgene had any capacity to increase *Btg1* expression in limbs at earlier embryonic stages, we carried out WISH in E9.5 embryos. As seen in the attached Figure R3, *Btg1* does not show any tissue-specific pattern at these early stages; instead, it seems to have an almost ubiquitous expression. We did not see any clear difference between wild-type and mutant embryos. Therefore, we ruled out the possibility of the transgene CS38-40 having a positive effect on *Btg1* expression in early limb buds (see Fig. R3; E9.5 embryos, scale bar 500μm) either because of the limb enhancer contained in the transgene, or associated to the transcription start site of lncRNAs *Hog* and *Tog* (whose extension is not covered by the transgene in any case). We have included the E9.5 WISH experiments (Fig. R3) as panel A in Figure S6.

Legend to Figure R3*.* Whole-mount in situ hybridization of *Btg1* mRNA in wild-type, heterozygous and homozygous transgenic E9.5 embryos. Two replicates are shown for each genotype. Scale bar 500μm.

*Regulatory effects 2:*

*Using publicly available data, such as chromatin ChIP-seq from the mouse ENCODE dataset, the authors could identify embryonic or adult tissues where the majority of regulatory elements is not cut off from Btg1 by the boundary insertion (kidney? heart? brain?). In these tissues (that could be obtained from the same crosses as used above) the loss of Btg1 expression should be less that in E12.5 limb buds. This data would strengthen the observation that the inserted boundary cuts off Btg1 from its major limb enhancers and the right Btg1 TAD boundary.*

As indicated by this referee, we analysed H3K27ac ENCODE datasets in E12.5 facial region, forebrain, midbrain, hindbrain, limb, heart and liver and E14 kidney (Figure R4). The distribution of acetylated peaks along the TAD is very heterogeneous in all tissues, yet we noticed that *Btg1* is covered by H3K27ac in all tissues (see grey bar). In addition, the putative limb enhancer (light blue bar) close to the right TAD border is also positive for this histone mark in all samples except in E12.5 liver.

Legend to Figure R4*.* UCSC tracks of H3K27ac data from the ENCODE project. Embryonic day and tissue are shown on the left. The position of the *Btg1* gene (grey bar), the insertion site of the TgN(38-40) (brown bar) and the putative limb enhancer (light blue bar) are shown. The distribution of wt TADs is depicted below the plots as dark blue horizontal bars.

As suggested by reviewer #3 (see below), we performed RT-qPCR in wild-type and mutant (in this case heterozygous for TgN(38-40) E12.5 limbs (in this case distal forelimbs as it seems to concentrate the limb positive signal in our WISH analysis, see Fig. 4), as well as in E12.5 livers (where the putative enhancer is not acetylated). These experiments showed that, in limbs, *Btg1* expression diminished upon insertion of TgN(38-40), while *Tbp1* (a gene we chose as an internal control) showed no change of expression. Conversely, the same two genes were not affected in liver cells. These results reinforced the idea that the segregation of this putative enhancer into a different TAD is at least partially responsible for the downregulation of *Btg1* in TgN(38-40) limbs. We have now incorporated the E12 limbs and liver H3K27ac profiles as part of Figure 4D and the RT-qPCR results (shown here as Fig. R5) as panel F of main Figure 4 in the manuscript.

Noteworthy, this analysis was carried out by using heterozygous mice, since the stocks had been frozen. Re-establishing the line to produce homozygous animals following crossing would have once again added at least half a year to the project, for a result which is already significant when using heterozygous limbs

Legend to Figure R5*.* RT-qPCR analysis of *Btg1* and *Tbp* (as internal control) in E12.5 limbs and livers from wild-type (wt, n=31) and heterozygous mutant embryos (*TgN(38-40)*/wt, n=28). *Actb* mRNA was used as a reference control. *P*-values are computed with Welch's t-test.

**Reviewer #3:**

*Defining the characteristics that are required for the formation of topological boundaries has received significant attention, including genetic experiments, biochemical analyses, computational, and modeling studies. Analysis of TAD boundaries has largely focused on use of deletions of chromosomal regions, or acute deletion of specific motifs such as CTCF binding sites, but relatively few studies have examined if the underlying DNA sequence can encode a boundary in different chromatin contexts.*

*In this manuscript the authors ask whether repositioning of a strong, CTCF-bound element from the HoxD cluster retains its potential to insulate chromatin when placed in an ectopic genomic context. The strength of this manuscript is in the novelty of performing this experiment using an in vivo context. However, as the boundary is moved to only one ectopic context, it cannot be determined if the results discussed herein can be extrapolated to additional regulatory sequences, for example boundaries with weaker insulation scores, or to different chromatin contexts.*

*Overall, the authors included excellent characterization of the integration site and the type of insertion event and have shown that the Tg mice of the large fragment acts similarly at the insertion site to its role in the endogenous environment. I think this work is sufficiently novel and would be of interest to the community, provided that the following comments are addressed:*

*Major points:*

*1. Title. I feel that the claim in the title is a bit misleading – there is only one integration site, so it is a bit of a stretch to conclude that the context doesn’t matter. In addition, the integrated sequence contains 4 CTCF reverse sites – would it still act as a boundary if it was in a context with no adjacent CTCF sites (for example in a compartmental domain or in another species where loop-extrusion loops are not so prominent such as drosophila)? To be clear, I don’t think that these experiments are required, I would just suggest to make the title more precise to avoid any misleading claims.*

We understand the concerns of this referee regarding the title of the manuscript and have modified it for: "INDUCTION OF A CHROMATIN BOUNDARY IN VIVO UPON INSERTION OF A TAD BORDER".

*2. Quantification of changes in Btg1 expression. Changes in Btg1 expression levels are difficult to quantify using WISH, it would be better to use qPCR as orthogonal approach, which could be used to examine the reduction in the context of both the homozygote and heterozygous animals.*

This concern was raised by both referees 2 and 3 and is addressed above (see Figure R5 and new Fig. 4F).

*3. Mechanism of Btg1 repression. One alternative to the hypothesis that Btg1 expression is downregulated due to the topological isolation of a putative enhancer is proximity spreading of a repressive mark such as H3K27me3. Indeed, the authors have previously shown that this region insertion is heavily decorated in Polycomb marks in its native context (Rodriguez-Carballo et al., 2019 – Figure 3). I think that this is an important conceptual distinction (linear spreading vs disruption of 3D landscape) and resolving it may enhance the conceptual novelty of the paper. H3K27me3 ChIP-seq in the Tg vs WT would resolve this question.*

We agree with referee 3 that region CS38-40 can recruit Polycomb in its native context and we also agree that discriminating between the effect of Polycomb/H3K27me3 nucleation/spreading and the disruption of the 3D landscape with respect to the downregulation of the *Btg1* gene would increase the scope of the paper. The changes we report in this study could be attributed either to topological alterations (as seems to be the case), or to the presence of the limb enhancer contained in CS38-40, or to a capacity of this region to recruit Polycomb.

Examining the latter possibility would involve a very substantial amount of work, while at the same time a recruitment of Polycomb to the exogenous CS38-40 being the cause for the downregulation of *Btg1* appears to be hardly plausible. First, the TgN(38-40) transgene shows very modest direct interactions with the *Btg1* gene unit itself, since we observe a reduced number of contacts only when using CS38 as a viewpoint. Second, it has been shown by us and others that non-expressed genes that are covered by Polycomb-associated chromatin marks tend to interact with one another, yet the dynamics and causality of this correlation regarding gene activity are not yet elucidated (Vieux-Rochas et al., 2015; Kundu et al., 2017; Cruz-Molina et al., 2017; Oksuz et al., 2018, amongst others).

*Minor Points:*

*- Please add mapping and QC stats for the Hi-C/4C experiments to better estimate the resolution*

We have now included a Supplementary Table (S5) with all the statistics regarding the Hi-C experiments presented in this article. We have also included Supplementary Tables (S6 and S7) compiling information about the 4C-seq mapped reads and the distribution of the 4C-seq fragments along the genome and the plotted areas.

*- Figure1: It would be great to also add the insulation score (similar to Figure4) to quantitatively show the loss of insulation upon deletion*

The insulation score has been added to Figure 1.

*- Figure3: Please show the CTCF ChIP signal in an unrelated control region (wt vs TgN(38-40), to ensure that the difference in intensity is indeed due to the presence of the extra CS38 site.*

We have now added a Supplementary Figure (new Fig. S4) displaying CTCF binding in three regions along different locations.

*- Figure4: it would be great to also show the H3K27ac ChIP-seq signal at the integration locus, in order to visualize the putative enhancer (instead of just mentioning it in the discussion).*

The H3K27ac wild-type track is now also included in Figure 4, in combination with ENCODE datasets.

*- Showing the insulation score in Fig 4 as an overlay between WT and the Tg line would make the comparison much easier for the reader.*

We agree that merging in the same panel the insulation scores from wild-type and mutant would facilitate the comparison. We have modified the figure accordingly, using the same colour codes as in the new panel of Fig. 1 (which also displays insulation scores).

**Have all data underlying the figures and results presented in the manuscript been provided?**

Large-scale datasets should be made available via a public repository as described in the *PLOS Genetics* [data availability policy](http://journals.plos.org/plosgenetics/s/data-availability), and numerical data that underlies graphs or summary statistics should be provided in spreadsheet form as supporting information.

Reviewer #1: Yes

Reviewer #2: Yes

Reviewer #3: Yes
